# A Case Report of Improved Palmoplantar Pustulosis following Periodontal Treatment and Possible Association with Diminished Systemic Subclinical Inflammation

**DOI:** 10.1155/2021/5548760

**Published:** 2021-10-19

**Authors:** Akiko Yamashita, Tomomi Sano, Misaki Iwashita, Fusanori Nishimura

**Affiliations:** ^1^Department of Periodontology, Division of Oral Rehabilitation, Faculty of Dental Science, Kyushu University, Fukuoka, Japan; ^2^Department of Cell Biology and Pharmacology, Faculty of Dental Science, Kyushu University, Fukuoka, Japan

## Abstract

Palmoplantar pustulosis (PPP) is a recurrent pustular dermatosis located on the palms and soles. Focal infection may exacerbate the symptoms of PPP, but the etiology is not fully clear. A 56-year-old woman with PPP was diagnosed with severe chronic periodontitis. Initial treatment for periodontitis combined with topical application of antibiotics and surgical treatment was performed. In this case, attention was paid to the relevance of systemic inflammation caused by periodontitis with the clinical symptoms of PPP. With periodontal treatment, the symptoms of PPP and periodontitis, high-sensitivity C-reactive protein (hs-CRP) level, and periodontal inflamed surface area (PISA) improved. This case highlights the importance of comprehensive dental examinations, including those for oral infections, such as periodontitis and other unrecognized sources of infection, and dental treatment in the overall management of PPP.

## 1. Introduction

Palmoplantar pustulosis (PPP) is a type of intractable, chronic, and recurrent pustular dermatosis located on the palms and soles [1]. Approximately 95% of patients are smokers at the time of onset [1, 2]. The disease usually takes a relapsing course and is often refractory to treatment [1]. PPP patients tend to have comorbidities, with an increased risk of autoimmune thyroid disease, type 2 diabetes, and so on [1]. PPP is a chronic skin disease that has a large impact on a patient's quality of life. Although the etiology of psoriasis, including PPP, has not been fully clarified, epidemiologic studies have suggested that the disease is often exacerbated by focal infections, such as tonsillitis, periodontitis, and metal allergy [3–5]. Currently, PPP is considered a type of psoriasis in most countries [6]. In contrast, it is considered a distinct disease entity from psoriasis in some countries because of the differences in genetic polymorphisms between the two [7]. Although many case reports have suggested a relationship between periodontitis and psoriasis, less than ten reports have described the relationship between periodontitis and PPP alone [2, 5]. According to a review, patients with periodontitis have a significantly elevated risk of psoriasis [8]. However, the role of the resolution of periodontal inflammation in PPP has not been well documented. Here, we report a case of PPP in a patient with suspected intraoral disease deteriorating factors who showed dramatic improvement in symptoms after periodontal therapy. The novelty of this report is that we particularly focused on inflammation and discussed the relationship between periodontitis and PPP from the standpoint of systemic inflammation, as assessed by the high-sensitivity C-reactive protein (hs-CRP) and related periodontal inflamed surface area (PISA).

## 2. Case Presentation

**Figure 1:**
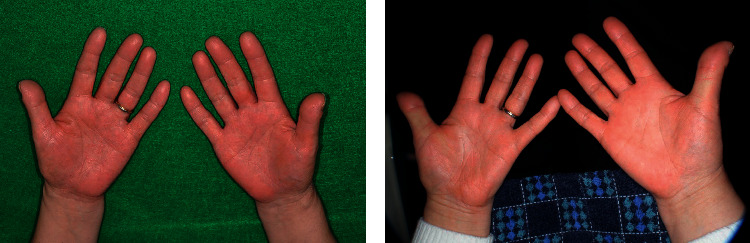
Appearances of the palm at (a) first visit and (b) 1 year after periodontal treatment. (a) Scaly erythemas with tiny pustules were scattered on the palm. (b) Marked improvement was observed after periodontal treatment.

A 56-year-old woman with PPP was referred to a dentist with suspected intraoral disease deteriorating factors (Figure 1(a)). She had discovered the symptom 2 years before on her palms and soles, smoking 10 cigarettes per day for 30 years. She did not have any arthralgia, suggestive of concomitant pustulotic arthro-osteitis (PAO). Since her symptoms did not improve spontaneously, she visited the dermatologist and was diagnosed with PPP and treated with topical steroids. The dermatologist also recommended that she undergo a patch test to diagnose a metal allergy, but it is physically difficult. Because her symptoms did not improve, she visited another doctor. The next doctor prescribed biotin, vitamin C, *Clostridium butyricum*, and oriental medicine (Asian ginseng and jumihaidokuto). Her symptoms eventually became refractory to treatment. The doctor noted her oral condition, and her medical history revealed oral discomfort, including gingival bleeding and swelling. Subsequently, the dermatologist suspected intraoral disease deteriorating factors and referred her to the Kyushu University Hospital Periodontology Clinic to investigate the possible association of PPP with her oral condition.

**Figure 2:**
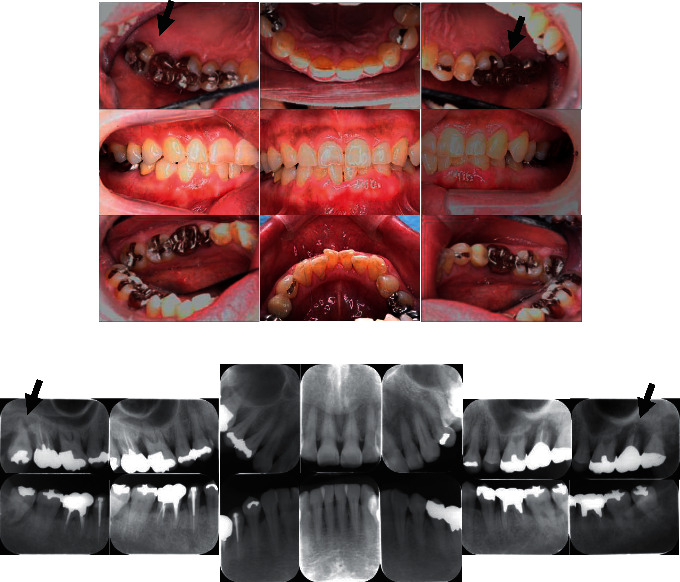
Appearance of the patient's periodontal tissues at the first visit (a) and radiograph of the periodontium (b). Note the severe alveolar bone resorption and so-called “floating teeth” due to severe periodontitis (b) and moderate gingival inflammation (a). “Floating teeth” were indicated by arrows. In addition, many metal restorations are observed.

**Figure 3:**
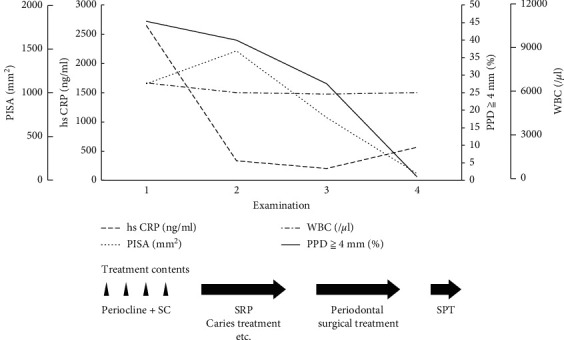
The course of dental treatment and the changes in hs-CRP levels, WBC, PISA value, and the ratio of PPD >4 mm. As periodontal treatment advanced, the value of inflammatory markers showed a decreased tendency.

Her medical records were unremarkable, except for severe chronic periodontitis. It was severe (stage IV and grade C) (Figures 2(a) and 2(b)). She had not received any prior comprehensive periodontal treatment. Upon her visit at our centre, we performed a regular examination and measured hs-CRP, white blood cell (WBC) count, and calculated PISA.  Figure 3 shows the results of the initial dental examination and the changes in each parameter according to the treatment. At the initial examination, the hs-CRP level, WBC count, PISA, and proportion of the probing pocket depth of over 4 mm were 2650 ng/mL, 6660/*μ*l, 1103.4 mm^2^, and 45.3%, respectively.

Because her hs-CRP level was relatively high, we first aimed to determine whether this elevation was due to periodontitis. We started the normal initial preparation with the topical application of antibiotics (minocycline hydrochloride ointment) to carefully observe the effects of anti-infectious periodontal treatment on PPP. We removed dental plaque and calculus and administered antibiotics in every pocket with a depth of >4 mm once a week for 1 month. A few months after the initiation of periodontal treatment, her palms, soles, and intraoral symptoms improved slightly. After initial preparation and reevaluation, we performed surgical treatment, including tooth extraction and correction of the irregular shape of the alveolar bone. Periodontitis showed further improvement 3 months after periodontal surgery (Figure 3). The symptoms of PPP gradually improved, and complete remission was observed in the supportive periodontal therapy (SPT) phase.  Figure 3 shows the changes in laboratory and clinical examination data. In the SPT phase, the hs-CRP level, WBC count, PISA, and proportion of the probing pocket depth of >4 mm were 565 ng/mL, 6020/*μ*l, 77.2 mm^2^, and 1.1%, respectively. The patient also showed improvement in PPP and periodontitis. After periodontal treatment, the patient continued SPT once every 3 months. In addition, although more than 2 years had passed after complete remission, no recurrence was observed (Figure 1(b)).

## 3. Discussion

PPP is considered a chronic inflammatory disease of the skin that severely impacts the patient's quality of life. Although the exact etiology of PPP is completely unknown, associations with thyroid disease, smoking, and focal infections, such as tonsillitis, periodontitis, and metal allergy, have been suggested [3–5]. A dermatologist recommended that the patient undergo a dental checkup because of suspected intraoral disease deteriorating factors. A dental examination revealed severe periodontitis.

Although the most contributory factor in the relationship between PPP and dentistry is a dental metal allergy, only 33% of the patients showed a positive response after metal removal from the oral cavity [9]. In addition, infection control has been reported to improve PPP^9^ symptoms. Periodontitis is the focus of focal infection.

With periodontal treatment, her PPP symptoms improved dramatically. After she was referred to our dental clinic, she stopped using the corticosteroid ointment. Furthermore, the doses of the other medications were unchanged. Smokers account for 95% of PPP patients [1]. Smoking is regarded as a strong risk factor for PPP [1]. Based on previous observations, smoking cessation is strongly recommended and may result in fewer pustules and lesser scales and erythema [1]. Furthermore, smoking exacerbates periodontitis and can affect the onset, progression, and recurrence of periodontitis [10]. It has been reported that hs-CRP is significantly higher in current smokers than in never and past smokers [11]. Therefore, we strongly recommended smoking cessation, but she has not quit smoking so far. There is a possibility that the continuation of oriental medicine and biotin might have improved her physical condition, leading to the improvement of PPP. However, along with the remission of inflammation, the symptoms dramatically improved as assessed by hs-CRP and PISA. The hs-CRP level is a highly sensitive low-grade inflammatory marker, and periodontitis is associated with elevated hs-CRP, decreasing with successful therapy [12]. On the other hand, PISA quantifies the degree of inflamed periodontal area, thereby quantifying the inflammatory burden posed by periodontitis [13]. It is beginning to be introduced as a new parameter of inflammation. Hs-CRP also decreased with periodontal treatment and showed a tendency to correlate with PISA in this case. It is considered that PISA at the second examination increased temporarily because the patient's tooth brushing condition before the examination was poor, and there were many points of bleeding on probing (BOP) at the time of periodontal tissue examination. The PISA increased as the BOP area expanded. In contrast, antibiotics were administered as needed during the period of aggressive periodontal treatment. However, antibiotics were not administered 3 months prior to the fourth examination. Therefore, the hs-CRP level in the fourth examination might have been higher than in the second and third examinations. As a result of additional examination after the fourth examination, hs-CRP was almost the same. It is considered that hs-CRP can be maintained within the normal range compared with the initial value. These findings suggest that severe periodontitis caused intraoral inflammation, increased PISA, and systemic inflammation, as indicated by increased hs-CRP. Furthermore, this inflammation participates in the deterioration of the PPP. Both conventional CRP and hs-CRP measure the same proteins. Conventional CRP measurements could not detect amounts below 0.1 mg/dL (1000 ng/ml). However, hs-CRP can measure trace amounts of CRP, which are normally undetectable. In this patient, the values at any examination were within the conventional CRP reference.

Previous studies have suggested that bacterial infection plays an important role in PPP. Some findings suggest that the immune responses to heat-shock proteins induced by chronically infected bacteria, such as periodontal bacteria, could also be etiological factors for PPP^3^. Some researchers have reported that the IL-23/IL-17 inflammatory pathway is important in the pathology of PPP [14]. Notably, several studies have reported that IL-17 is associated with periodontitis, psoriasis, and other immune-mediated inflammatory diseases [15, 16].

Dental metals, focal infections, and low-grade inflammation are possible deteriorating factors that influence the pathology of PPP. In this case, the symptoms of PPP improved, even without the removal of dental metals. Therefore, intraoral focal infections and inflammation should be carefully considered. Removing oral infectious lesions may become first-line therapy in dental and medical treatment because controlling infections is much easier and more cost effective than replacing restorative materials. Some reports have indicated that intraoral and pharyngeal focal infections are closely related to the onset of PPP [5, 17]. In this regard, calculating PISA as an indicator of local intraoral inflammation and measuring hs-CRP as an indicator of low-grade systemic inflammation would be useful markers to assess the contribution of periodontitis to the deterioration of PPP.

In conclusion, this case highlights the importance of comprehensive dental examinations, including those for dental infections, such as periodontitis, dental abscesses, and other unrecognized sources of infections, dental treatment, and assessment of low-grade inflammatory markers as hs-CRP in the overall management of PPP.

## Data Availability

The data used to support the findings of this study are included within the article.
